# Pulmonary hypertension in South Africa – at last some original research!

**DOI:** 10.7196/AJTCCM.2018.v24i4.233

**Published:** 2018-12-20

**Authors:** A Goolam Mahomed

**Affiliations:** Louis Pasteur Private Hospital and Mediclinic Pretoria Heart Hospital, Pretoria, South Africa

## Editorial

Very little information is available in South Africa (SA) on pulmonary vascular disease. The article on pulmonary hypertension (PH) by Davies-van Es *et al*. ^[1]^ in this issue of the AJTCCM is one of the first attempts to rectify this, and is highly welcome. The Groote Schuur Hospital Pulmonary Hypertension Clinic is one of very few specialised units in SA in the management of pulmonary vascular disease. Even in a specialised unit such as this one, the article highlights the deficiencies that exist in the management of PH in SA.

Many patients in the study lacked documented tests, including echocardiography, although this may be due to the retrospective nature of the study. The work-up is particularly deficient in doing cardiac catheterisation, and also in vascular reactivity tests. Cardiac catherisation was done in <50% of the patients. Vasoreactivity testing was predominantly done using intravenous nitrates, although this is not the recommended test for vasoreactivity. The authors suggest that the cost of doing the recommended vasoreactivity testing is prohibitive in the SA context, and also the availability of the agents becomes an issue.

An interesting aspect of the study is the high number of patients who had HIV-associated pulmonary arterial hypertension. This is not unexpected, given the high prevalence of HIV in the SA community, but I believe that this is one of the first studies that actually documents this in a PH clinic. A previous study in SA looked at the incidence of HIV-associated PAH in a cardiac clinic in Soweto, and reached an incidence of 8.1%.^[2]^ Pooled data from three studies in Africa, including the Soweto study, suggested a prevalence of 14%.^[3]^

The study also highlights the lack of availability of specific PH therapy in SA, which has been lamented previously as well.^[4]^ Only five patients at the clinic were on specific PH therapy, and in the main this was limited to sildenafil. Macitentan is now also available in SA, but it will be a while before this is also used, owing to funding limitations.

There was a relatively high incidence of smoking in this cohort, and this should be explored in detail in further studies, although it may only be a reflection of the incidence of smoking in the community.

Overall, this study is a welcome contribution to our understanding of PH in SA, and we hope it will be a stimulus for much more research, including prospective studies.
